# *WWOX* Tumor Suppressor Gene in Breast Cancer, a Historical Perspective and Future Directions

**DOI:** 10.3389/fonc.2018.00345

**Published:** 2018-08-28

**Authors:** Karolina Pospiech, Elzbieta Płuciennik, Andrzej K. Bednarek

**Affiliations:** Department of Molecular Carcinogenesis, Medical University of Lodz, Lodz, Poland

**Keywords:** WWOX, breast cancer, tumor suppressor, carcinogenesis, cancer progression

## Abstract

The *WWOX* tumor suppressor gene is located at 16q23. 1–23.2, which covers the region of FRA16D—a common fragile sites. Deletions within the *WWOX* coding sequence are observed in up to 80% of breast cancer cases, which makes it one of the most common genetic alterations in this tumor type. The *WWOX* gene is known to play a role in breast cancer: increased expression of *WWOX* inhibits cell proliferation in suspension, reduces tumor growth rates in xenographic transplants, but also enhances cell migration through the basal membrane and contributes to morphological changes in 3D matrix-based cell cultures. The WWOX protein may act in several ways, as it has three functional domains—two WW domains, responsible for protein-protein interactions and an SDR domain (short dehydrogenase/reductase domain) which catalyzes conversions of low molecular weight ligands, most likely steroids. In epithelial cells, WWOX modulates gene transcription through interaction with p73, AP-2γ, and ERBB4 proteins. In steroid hormone-regulated tissues like mammary gland epithelium, the WWOX SDR domain acts as a steroid dehydrogenase. The relationship between WWOX and hormone receptors was shown in an animal model, where WWOX(C3H)+/–mice exhibited loss of both ER and PR receptors. Moreover, in breast cancer specimens, a positive correlation was observed between *WWOX* expression and ER status. On the other hand, decreased *WWOX* expression was associated with worse prognosis, namely higher relapse and mortality rates in BC patients. Recently, it was shown that genomic instability might be driven by the loss of WWOX expression. It was reported that *WWOX* plays role in DNA damage response (DDR) and DNA repair by regulating ATM activation through physical interaction. A genome caretaker function has also been proposed for *WWOX*, as it was found that WWOX sufficiency decreases homology directed repair (HDR) and supports non-homologous end-joining (NHEJ) repair as the dominant DSB repair pathway by Brca1-Wwox interaction. In breast cancer cells, *WWOX* was also found to modulate the expression of glycolysis pathway genes, through hypoxia-inducible transcription factor 1α (HIF1α) regulation. The paper presents the current state of knowledge regarding the WWOX tumor suppressor gene in breast cancer, as well as future research perspectives.

## *WWOX* tumor suppressor gene—discovery and characteristics

The *WWOX* tumor suppressor gene was identified and cloned in the year 2,000 following research on the 23.1–23.2—region of long arm of chromosome 16. This region was also identified as FRA16D: one of the common chromosomal fragile sites (1). For a long time this region was of particular interest, because of the high incidence of loss of heterozygosity (LOH); this was first observed in prostate cancer (2, 3), and later in several other tumor types, including liver cancer (40% LOH) (4–7), ovarian cancer (8), ductal breast cancer *in situ* (DCIS) (about 80% LOH) (9), sporadic breast cancer (10), extrahepatic bile ducts (11), esophageal squamous cell carcinoma (12, 13), non-small lung (14), pancreas (15, 16), multiple myeloma (17), thyroid (18), glioblastoma multiforme (19), and Wilms tumors (20–30% LOH) (20).

A significant reduction or lack of expression of WWOX gene was observed mainly in breast cancer, but also (amongst other) in esophageal squamous cell carcinomas (12), non-small lung cancers (14, 21), pancreatic tumors (15, 16, 22) prostate, (23), gastric, ovarian (24), thyroid (18, 25), and bladder (26) cancers.

The highest expression of *WWOX* was observed in normal testis, prostate, and ovary tissues, while considerably lower expression was observed in the colon, small intestine, thymus, and spleen. The fact that that highest expression was observed in hormone-regulated tissues also characterized by the presence of an enzymatic dehydrogenase/reductase domain indicated that its protein product may be involved in the metabolism of steroid hormones (27). Nunez MI et al. carried out a wide range of tissue array analysis of *WWOX* expression in human normal tissues, derived from more than 30 organs. Highest expression was found in the fallopian tubes, ovaries, mammary gland epithelial cells, endometrial, prostate, testes, liver, stomach, salivary glands, adrenal gland, thyroid, parathyroid, pituitary, cerebellum, and brain cells. In contrast, no expression was observed in fatty tissues, connective tissues, lymphoid tissues, myelin structures or blood vessels. Several recent RNASeq analyses of normal human tissues correlate previous findings, revealing highest *WWOX* expression in thyroid tissue, testis and brain–particularly the cerebellum (28–30).

## WWOX protein versatile functions

Further studies revealed that *WWOX* gene encodes a protein of 414-amino acids (46 kDa) localized mainly within the Golgi system and cytoplasm. At that time, it was the only known protein containing both a short-chain dehydrogenase/reductase (SDR) central domain and two WW domains at the NH_2_ (1, 31). Studies of *WWOX* expression identified several alternative transcripts. Such aberrant transcripts encoding truncated proteins (predominantly devoid of oxidoreductase-coding sequence) were found not only in the breast cancer cell lines and tumor samples (31), but also in various cancerous tissues; however, no other form of WWOX truncated protein was found (31–34).

Interestingly, it was shown that only the first WW domain binds to PPXY motifs in a physiologically relevant manner, whereas the second does not exhibit affinity toward any WW-domain targets. This has been attributed to the double substitution of chemically distinct amino acids located within the binding pocket of the WW2 domain. Also while the first WW domain was found to be unfolded just until ligand binding, the second adopts a fully structured conformation and was also found to help in the stabilization and binding of the ligand to the WW1 domain. All these results suggest that the WW2 domain of WWOX protein serves as a chaperone to augment binding of WW1 domain within WWOX to PPXY motifs of WBP1 and WBP2 proteins (35–37), along with other partner proteins, such as ERBB4 (38).

In Eukaryotes, proteins with a WW domains are involved in various processes associated with cellular signaling, transport of proteins, transcription and RNA processing. Based on pull-down assay, Abu-Odeh et al. published the list of above 200 potential WWOX protein partners (37), which included several transcription factors, such as P73 (37, 39), AP2-γ (40), c-Jun (41), DVL-2 (37, 42), RUNX2 (43), SMAD3 (44), and GLI-1 (45). Binding with WWOX causes their sequestration in the cytoplasm (or in the nucleus, in the case of RUNX2), thereby inhibiting their trans-activating function in the nucleus. WWOX protein also has the ability to bind to the cytoplasmic intracellular part of the ErbB4 receptor, which results in the inhibition of Yap protein-mediated transcription co-activation in the nucleus (46). In addition, studies have found the WWOX protein to bind to ezrin (47)—an actin-binding protein responsible for driving cell migration which may affect remodeling of the actin cytoskeleton (48). Recently, it was also confirmed that WWOX physically interacts with the ITCH protein—an E3 ubiquitin ligase which ubiquitinates Lys-63 of WWOX leading to its nuclear translocation. Lys-63-linked poly-ubiquitination is known to play a role in the DNA damage response; therefore, WWOX translocation to the nucleus might be associated with increased cell death. WWOX nuclear translocation was also formerly described for RUNX2 (43) and p53 (49) -WWOX protein interactions. Although several WWOX interactions have been identified, the exact function of the WWOX protein is not yet fully understood.

WWOX amino-acid sequence analysis identified two most conserved features of SDR proteins: the coenzyme NAD(H)/NADP(H) binding site and the potential substrate binding site (50, 51). Sequence similarity studies have classified WWOX protein as steroid dehydrogenase (1, 52), more precisely, 17β-hydroxysteroid dehydrogenase (53, 54). However, attempts at characterizing WWOX protein enzymatic activity have proved to be unsuccessful due to inability to purify the protein without loss of activity. This may suggest that *in vivo*, the WWOX protein is only present in liaison with other cell proteins which should be substituted in bacterial expression systems (55).

Animal-model based studies strongly support the hypothesis that the WWOX protein indeed participates in sex-steroid metabolism (56). Aqeilan et al. confirmed the importance of WWOX in steroidogenesis and proper gonadal function in knock-out mice (KO). The reduction or absence of WWOX was associated with Leidig cell formation failure, untraceable status of testosterone in serum, decreased theca cell proliferation and tinier ovarian follicles in KO mice. Additionally, the absence of WWOX was found to lead to differential expression of 15 steroidogenesis-associated genes (57). Other studies have also established the importance of WWOX in the alteration of HDL and lipid metabolism (58, 59).

## WWOX in DNA damage response, and genomic stability

The relationship between the variable expression of DNA damage response proteins (p53, BRCA1, γH2AX, pChk2), DNA damage-sensitive tumor suppressors (WWOX, FHIT), WWOX-interacting proteins (Ap2- α and γ, ERBB4), cancer subtype, and clinical factors was tested in a tissue microarray analysis of 479 cases of breast cancer samples. BRCA1 nuclear expression was associated with FHIT and WWOX, and the absence of WWOX was strongly associated with loss of FHIT expression and cytoplasmic ERBB3. Additionally, a strong correlation was observed between FHIT expression and two WWOX partner proteins: cytoplasmic ERBB4 and Ap2α. In the multifactor model, triple negative breast tumors showed significantly reduced expression of WWOX. Furthermore, the expression of WWOX and ERBB4 was significantly lower in tissues derived from lymph node matched metastases than primary breast cancer tissues. The authors suggest that the loss of the signal pathway in which WWOX is involved contributes to lymph node metastasis by allowing detached cancer cells to survive without contact with the basal membrane (60).

An analysis of the significance of WWOX in DNA damage response (DDR) and DNA repair in MCF7 breast cancer cells revealed that induction of DNA double-strand breaks (DSBs) by ionizing radiation resulted in transient twofold elevation of WWOX mRNA level after 10 min exposure, which subsequently returned to baseline after 1–2 h. To exclude line specificity, this was further confirmed at the protein level, not only for MCF7 but also for primary mouse embryonic fibroblast (MEF), human embryonic kidney cells (HEK293), and osteosarcoma LM7 cells, both employing IR, as well as radiomimetic drug neocarzinostatin (NCS). Moreover, loss of WWOX expression in MCF7 breast cancer cells was found to be associated with the increased DSB level following DNA damage, highlighting the role of WWOX in genomic stability. The absence of WWOX leads to diminished activation of checkpoint kinase ATM, inefficient induction and maintenance of γ-H2AX foci, and defective DNA repair (61). Upon DNA damage, ATM stimulates the activity of ITCH (E3 ubiquitin ligase) (62), which in turn enables WWOX Lys-63 ubiquitination, thus promoting WWOX protein translocation to the nucleus. WWOX in the nucleus was found to interact with ATM, enhancing its monomerization and activation in a positive feed-forward loop manner (61).

The same research group underlined the importance of WWOX upon DNA single-strand breaks (SSBs) checkpoint activation. It turned out that introducing SSBs resulted in raised WWOX protein level and its accumulation in the nucleus, whereas WWOX depletion was associated with reduced activation of ATR checkpoint proteins and increased chromosomal breaks. The molecular mechanism of WWOX regulation of ATM activation was observed as described previously (61). On the other hand, the correlation between the inhibition of ATM and reduction in activity of ATR checkpoint kinases indicates that the effect of WWOX on ATR is influenced by ATM (63).

Recently Schrock et al. employed human embryonic kidney 293T cells and MDA-MB-231 breast cancer cells to describe interaction of Wwox and Brca1 proteins. The latter is known to mediate homology directed repair (HDR) of DSBs. Unlike for Wwox-ATM, the interaction of WWOX with Brca1 did not seem DNA-damage dependent; it was therefore suggested that at sufficient levels, Wwox might compete with various Brca1-interacting proteins important for HDR. In a model based on Rad50 protein, which is a component of the MRN complex interacting with Brca1, Wwox competes with it for binding to Brca1 and consequently impairs end resection. Thus in cancerous cells exhibiting depletion of Wwox, an important inhibitory step would be absent, which might result in enhanced end resection and HDR repair, finally allowing the cells to survive DNA damage-inducing cytotoxic treatments. Indeed it was found that loss of Wwox protein expression contributed to radiation and cisplatin resistance in mouse embryonic fibroblasts (MEFs) and human MDA-MB-231 breast cancer cells, which in turn might be associated with cancer recurrence and poor clinical outcome. Both human breast MCF10A and mouse MEF cells lacking Wwox exhibited enhanced survival upon DSBs inducement by means of ionizing radiation and bleomycin treatment. MDA-MB-231 cancer cells, which survived IR recurred faster in a xenograft model of irradiated breast cancer cells. Also Wwox-deficient MDA-MB-231 cells revealed shorter tumor latencies than the cells expressing Wwox. In a group of brain cancer patients treated with radiation, Wwox deficiency significantly correlated with shorter overall survival times: data obtained from Repository of Molecular Brain Neoplasia Data (REMBRANDT).

Thus according to the model proposed, Wwox might influence the choice of DNA DSB repair pathway by suppressing HDR by interacting with Brca1, and at the same time enhancing non-homologous end-joining (NHEJ) repair. It was also suggested that WWOX might serve as a genome caretaker, with its function based on Brca1-Wwox interaction in turn supporting NHEJ as the principal DSB repair pathway in Wwox-expressing cells (64).

## Biological and clinical implications of WWOX in breast cancer

### WWOX genetic changes—*in vivo* and *in vitro* studies

Suppressive properties of *WWOX* gene were widely studied and confirmed by various research groups. *In vivo* studies showed that transduced MDA-MB-435 breast cancer cells exhibiting elevated WWOX expression injected into mammary gland of athymic mice delayed and inhibited tumorigenesis and slowed tumor growth rates compared to xenografts formed after control injections with unmodified MDA-MB-435 cells. Moreover, the tumors formed by MDA-MB-435/vector transduced cells were found to be about 10-fold larger than tumors formed by cells transduced with the *WWOX* gene (31). A similar inhibition of tumor growth in athymic mice after injection of *WWOX* transduced cells was also observed for other cancer cell lines, including pancreatic cancer cell line- Panc-1 (15), lung cancer - A549, H1299 H460 (65), prostate cancer–DU-145 (66), and ovarian cancer–PEO1 (67).

*In vitro* studies confirmed the suppressive characteristics of the WWOX gene. Soft agar growth assay showed the restoration of *WWOX* gene expression in breast cancer cell lines MDA-MB-453, T47D (31), BT-474, MDA-MB-231 (68, 69) and HCC1937 (69) significantly reduced their ability to anchor independent growth. Similar observations were reported for other *WWOX*-transduced cell lines from other cancers: pancreatic–AsPc1 and Panc1 (15, 16), prostate–DU145 (66), lung–A549, H460 and H1299 (65), and ovarian–A2780 (70).

Additionally, induction of apoptosis was observed through activation of the internal caspase cascade in the ectopically *WWOX*-expressing breast cancer (Ad-WWOX MDA-MB- 231) and lung cancer cell lines (Ad-WWOX A549, Ad-WWOX H460) (65, 69).

Another cancer-specific feature is the migration of cancer cells through a basal membrane, which is a basis of tumor aggressiveness. Surprisingly, contrary to animal studies, where WWOX inhibits tumorigenesis, elevated transcription of *WWOX* in MDA-MB-231 breast cancer cells was found to escalate migration through the basal membrane (Matrigel test), suggesting increased invasiveness (68). On the other hand, a morphogenesis test in Matrigel revealed MDA-MB-231 cells transduced with *WWOX* to display branched structures, compared with the spherical tumor-like structures of control cells. This results suggest that the *WWOX* gene might be involved in normal mammary tissue development (68). In ovarian cancer cell lines it was also observed that the restoration of WWOX modulated interaction of tumor cells with the extracellular matrix (67).

Gene function can be effectively investigated using gene knock-out experiments. A 2011 study based on WWOX(C3H)^+/−^, a WWOX-heterozygous C3H mammary tumor-susceptible genetic background mouse strain which resembles possible malignant transformation in humans, found that 50% of female mice formed breast cancer compared to 7% in the control WWOX(C3H)^+/+^. In most of the WWOX(C3H)^+/−^ tumors, loss of estrogen and progesterone receptors was also observed. Furthermore, cDNA array expression analysis of normal murine breast tissue and heterozygous WWOX(C3H)^+/−^ tumors was carried out, and identified 292 genes of significantly variable expression. Most identified genes turned out to be involved in cellular movement, signaling and interactions, cellular development, growth, proliferation, and cell death (71). Initial attempts of developing adult WWOX^−/−^ knock-out mice turned out unsuccessful, as animals were smaller and died within 4 weeks. Moreover, WWOX^−/−^ mice developed spontaneous osteosarcomas, whereas WWOX^−/+^ mice squamous lung carcinomas. Compared to wild-type animals (WWOX^+/+^), neoplasms were five times more frequent. In addition, the presence of WWOX protein in tumors of WWOX^−/+^ animals, suggests a predisposition to malignancy as an effect of haploinsufficiency (57). WWOX^−/−^ mice showed also severe metabolic defects, which affected proper bone formation (43).

Interestingly, in 2012 Ferguson et al. for the first time described tissue specific targeted ablation of *Wwox* gene in adult mouse tissues. Their MMTV-Cre mouse model exhibited significant down-expression of *Wwox* in the mammary epithelium without any adverse effect on survival. What is more, *Wwox* deletion did not affect tumorigenicity, nor did haploinsufficiency affect the mammary gland phenotype: It was concluded that *Wwox* might not be a classical tumor suppressor gene, but that rather loss of *Wwox* expression is associated with tumor progression. Nevertheless, *Wwox* knockdown in a mouse model resulted in impaired mammary branching morphogenesis (72). Similar observations, were made on a breast cancer cell line, where ectopical overexpression of *WWOX* gene in MDA-MB-231 breast cancer cells changed cell growth in Matrigel from tumor-like to branched structures, which resembled normal mammary duct formation (68).

Great efforts have been made to reveal the biological functions of *WWOX* and identify the signaling pathways associated with its expression, not limited to breast cancer. For *Wwox* KO MMTV-Cre mice, significant deregulation of the genes involved in various cellular processes was observed in the mammary epithelium. Gene ontology enrichment analysis of the Biological Processes GO category identified WWOX-associated expression of Wnt signaling pathway genes, including significant upregulation of *Wnt5a*, which is also transcriptional target of the TGFβ/SMAD signaling pathway, skeletal system development/bone morphogenesis, genes associated with tissue remodeling, and cell migration as well as adhesion-related genes (for instance *Timp2* and *Timp3* upregulation) (72). An interesting strategy for elucidating WWOX function was employed by Aldaz et al. Their study used a Multiexperiment Matrix bioinformatics tool to identify the top 100 genes positively-correlated with WWOX and the top 100 negatively-correlated genes based on approximately 4,800 samples of both normal and tumor tissues, as well as breast cancer cell lines, obtained from breast datasets. Among the top enriched biofunctions, the following were identified: “regulation of mammary gland morphogenesis and branching,” “coenzyme A metabolic process,” “WNT signaling pathway,” “senescence/autophagy” and “fat cell differentiation” (73); these results are consistent with those of previous studies.

### Clinical significance of WWOX in breast cancer

As mentioned above, breast cancer deletions within the common chromosomal fragile site FRA16D are observed in more than 80% cases, which makes changes in the WWOX coding region the most common genetic alternation in breast cancer (9). However, LOH is not the only mechanism responsible for *WWOX* downregulation: hypermethylation (69, 74, 75) and some infrequent point mutations within the coding region of *WWOX* (76) have also been found to be involved in breast carcinogenesis and cancer progression. In addition, rare homozygous deletions have also been observed for lung, ovarian, pancreatic (34) and colon cancers (77), WWOX protein degradation has been observed as a result of ubiquitination in prostate cancer (78), and hypermethylation in gastric (6, 79), pancreatic (16, 80), bladder (74), lung (74, 81, 82), and prostate (66) tumors.

The first cell line-based *WWOX* gene expression study related to breast cancer found HME-87 expression to be elevated in all eight tested cell lines compared to normal epithelial breast cells. Interestingly, the studied cell lines demonstrated wide variations in expression, ranging from relatively low levels in T47D, MDA-MB-435, and MDA-MB-231 to significant over-expression in ZR75-1, MCF-7, and MDA-MB-361 (1, 31, 83). This diversity in WWOX expression in different breast cancer cell lines was confirmed by Driouch et al. who also showed decreased expression in breast cancer tumors (32).

Immunohistochemical studies also found 63.2% of 97 invasive breast cancers to demonstrate a reduction in WWOX protein level. In addition, in 32.9% of these cases also demonstrated reduced expression in normal breast tissue, and correlation between reduced expression and a higher degree of tumor stage was shown (*P* = 0.033) (84). Similarly, Nunez et al. report high WWOX protein levels in normal human epithelial breast tissues, but very low or no expression in 33% of DCIS and 59.6% of invasive breast cancer cases (85). Reduced expression of WWOX was also observed in immunohistochemical study of 44 DCIS tumors (68.2%), 31 DCIS tumors adjacent to invasive breast cancer (54.8%), 30 cases of invasive breast cancer (61.3%), 39 normal tissues adjacent to DCIS tumors (56.4%), and 30 healthy tissues surrounding DCIS tumors adjacent to invasive breast cancers (29%). Moreover, reduced expression was observed more frequently in tissue adjacent to invasive breast cancer of a higher DCIS tumor stage (*P* = 0.004) (86). Another analysis of 267 breast cancer cases revealed an association between lower WWOX expression, the basal breast cancer subtype (*P* = 0.01) and shorter relapse-free survival (DFS), although its level did not correspond to patient overall survival (OS) (86).

*WWOX* was also proved to be a potent prognostic marker in breast cancer. In 2010 Pluciennik et al. assessed *WWOX* and three other genes (*ESR1, CDH1, BAX*) as a markers of favorable prognosis in nearly 120 BC patients. Along with *KTR5, KRT14, KRT17, CCNE1, BCL2*, and *BIRC5* genes, found to be associated with unfavorable prognosis, those 10 genes constituted an algorithm dividing patients into statistically significant groups of diverse disease-free survival times (*p* = 0.0056). Additionally, after division of the patients according to ER status, the algorithm was only well diversified in the group of ER-patients (*p* = 0.0039) (87).

The prognostic value of WWOX expression in breast carcinomas, was also assessed by Aldaz et al. 73, when he and his co-workers employed publicly available gene expression microarray data sets and Kaplan–Meier Plotter tool, in order to stratify breast cancer patients according to high and low WWOX expression. The findings revealed low WWOX expression to be associated with shorter relapse-free survival times in breast cancer patients (*n* = 3259). Additionally, while this trend was confirmed in all breast cancer subtypes, it was definitely more evident in luminal B and basal-like subtypes than luminal A (73).

### Association of WWOX and ER status and chemotherapy effectiveness in breast cancer

The first report of a correlation between WWOX and estrogen receptor status obtained by a immunohistochemical study of WWOX in 97 archived breast carcinoma specimens in relation to various patient and tumor characteristics by Guler et al. It was found that reduced WWOX staining was associated with less favorable ER status (*P* = 0.033). Lowered WWOX expression was observed in 56.3% ER-negative tumors and < 25% ER-positive tumors, while normal WWOX staining correlated with negative or nearly negative ER status only in 30.4% of cancer cases (84).

Nunez et al. identified a correlation between WWOX expression and ER status. They compared WWOX protein level with patient clinico-pathological profile in a group of 16 human normal breast epithelium samples, 15 DCIS tumors and 203 invasive breast cancer cases. They observed that 27% of estrogen-positive breast carcinomas were negative for *WWOX* expression, compared with 46% for ER– cancers (*p* = 0.0054). In addition, when combining *WWOX*–deficient and nearly-negative cases, the difference became even more substantial, with 51% of ER+ cases and 73% of ER– cases being recognized (*p* = 0.003) (85). Similar correlations were also observed in other studies. The level of *WWOX* expression correlated with ER and PR status in a quantitative real-time RT-PCR study of 132 breast cancer cases: significantly higher WWOX expression was observed in ER+ tumors compared to ER– tumors, as well as in PR positive cancers compared to PR negatives, and in ER+PR+ tumors compared to ER–PR– cases (88). A strong positive relationship was found between WWOX expression and ER (*p* < 0.001) or PR (*p* = 0.001) by immunostaining of tissue microarrays constructed from 837 breast cancer blocks (89).

WWOX has been also described as a promising marker of chemotherapy effectiveness. Scientists observed 4.6-times greater probability of tamoxifen resistance in patients with reduced expression of WWOX (*n* = 89 breast cancer patients). In addition, the expression of WWOX was found to be a better marker of tamoxifen resistance in high-risk patients than progesterone receptor level (90). Also, an *in vitro* study showed that a reduction of WWOX resulted in diminished ER levels and diminished tamoxifen sensitivity (71). The association between WWOX expression and sensitivity of tamoxifen treatment was finally confirmed on tissue microarrays employing 912 randomized breast cancer tissues. In patients treated with tamoxifen, there was a significant correlation between high WWOX expression and a lowered risk of recurrence, indicating that WWOX might be a potential marker of tamoxifen effectiveness (91).

### WWOX in bone metastases of breast cancer and metabolism

In 2013, Matteucci et al. investigated WWOX, E-cadherin and TAZ protein levels, with the intention of deciphering the contribution of Hippo-related pathways in bone metastasis from breast cancer. Interestingly, the findings indicated elevated WWOX levels in bone metastases prevalently at plasma membrane/nuclei level and in cytosolic-perinuclear areas (*n* = 15), while being almost absent in primary invasive ductal breast carcinoma tissues (*n* = 6; two specimens matched) (92–94). Moreover, the adjacent mammary tissues showed a WWOX signal (93, 94). *In vitro* studies showed that WWOX and E-cadherin were higher in bone-metastatic 1,833 cells when compared with parental-MDA-MB-231 cells, while being elevated in non-invasive MCF-7 breast carcinoma cells. Knocking-down endogenous WWOX reduced invasion and E-cadherin expression in 1,833 cells, whilst its overexpression enhanced E-cadherin transactivation and protein level in the 1,833, but not in MDA-MB-231 cells, increasing also metastatic-cell migration. It was concluded that in 1833 cells, WWOX expression varies with DNA-methylation state and hypoxic conditions (92, 93), as endogenous levels of WWOX protein in the 1833 cells was regulated by DNA methyltransferases. Long-term exposure to the inhibitor 5-azacytidine caused WWOX downregulation and treatment of 1,833-xenograft mice with another inhibitor of DNA methyltransferases, 5-aza-2′-deoxycytidine, prolonged mouse survival and increased WWOX expression both in cytosol and nuclei. WWOX and TAZ were found to activate Hypoxia inducible factor-1 (HIF-1) binding to E-cadherin promoter, while PPARγ receptor (Peroxisome proliferator-activated receptor γ) mediated in E-cadherin transactivation supporting WWOX and preventing TAZ functions (92–94). Further research revealed that by influencing E-cadherin expression, Wwox contributes to mesenchymal-epithelial transition (MET) and colonization of bone metastasis from breast carcinoma (94).

HIF-1 was found to be a major regulator of oxygen homeostasis and of aerobic glycolysis in cancer (Warburg effect), and HIF-1α its inducible subunit (95). It was found that WWOX regulates the expression of glycolysis-related genes through HIF-1α (96).

At this point it is scarcely possible not to mention the emerging role of WWOX in the metabolic state of the cells, which is of particular importance given the fact that reprogramming of energy metabolism was recently added to the list of hallmarks of cancer cells (97). During glycolysis, normal cells convert glucose to pyruvate in the cytosol, which is followed by complete oxidation of pyruvate to CO_2_ through the tricarboxylic acid (TCA) cycle and then oxidative phosphorylation in the mitochondria, under aerobic conditions; under anaerobic conditions, the pyruvate is metabolized to lactate. However, cancer cells convert most glucose to lactate even in aerobic conditions, diverting glucose metabolites from energy production to anabolic process to accelerate cell proliferation. This state has been termed the Warburg effect, after the first researcher to describe the phenomenon, or as aerobic glycolysis (98).

**Figure 1:**
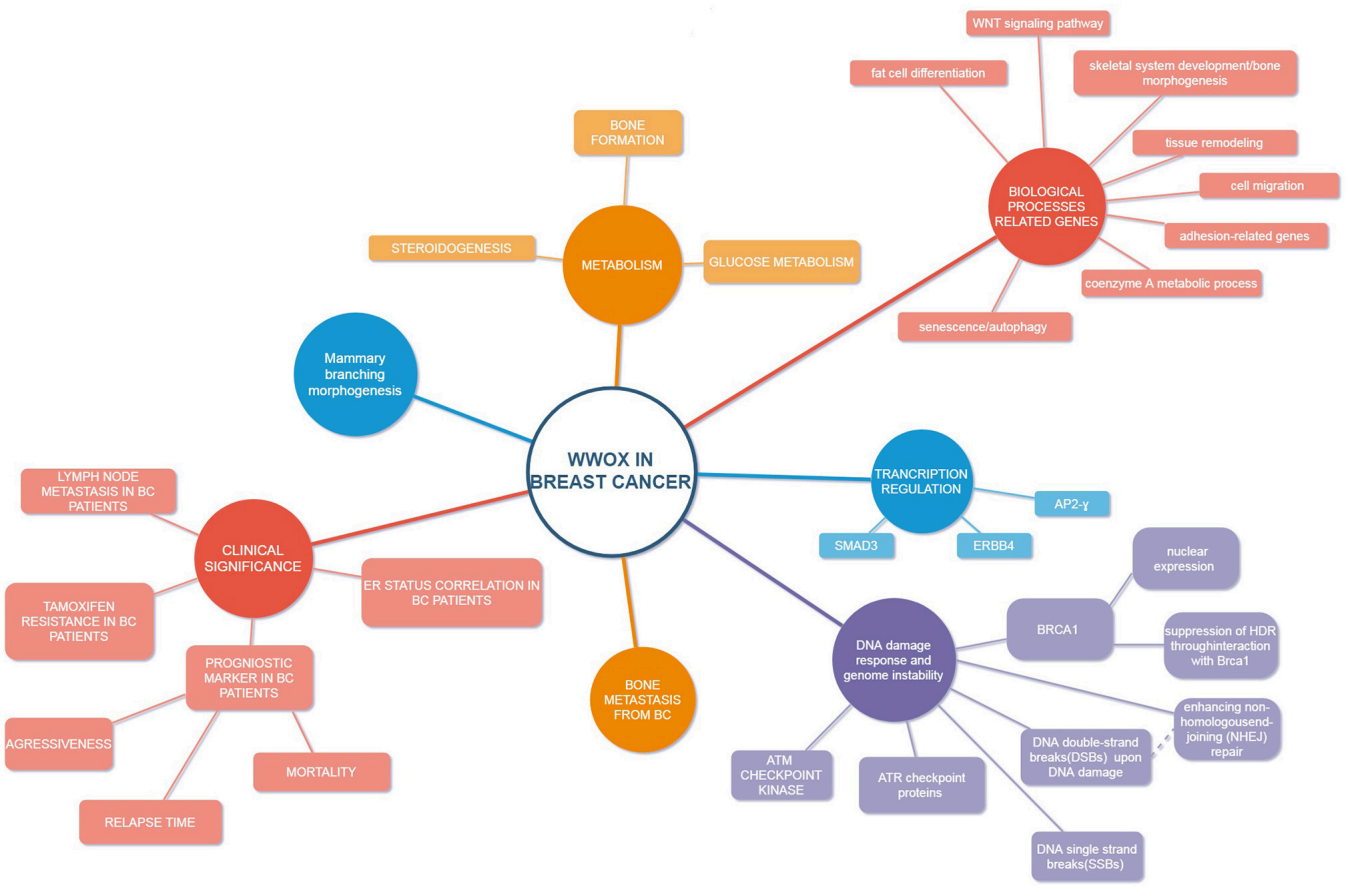
Schematic model of WWOX in breast cancer.

As mentioned above, Abu-Remaileh and Aqeilan (96) reported that WWOX, physically and functionally interacts with HIF1α and regulates its transactivation function both *in vitro* and *in vivo*: *WWOX* KO mice exhibit elevated levels of serum lactic acid. The loss of *WWOX* resulted in activation of glycolysis in mouse embryonic fibroblasts (MEF) from KO embryos, compared with fibroblasts from *WWOX*-wild type embryos. Importantly, genetic or pharmacologic depletion of HIF1α was able to reverse WWOX-mediated phenotypes associated with its loss, including tumorigenesis. In addition, breast cancer tissue microarrays have shown that in breast cancer samples, WWOX expression is inversely correlated with levels of the glucose transporter GLUT1, which is known to be a direct target of HIF1α. This again highlights the modulatory role of WWOX in cancer metabolism (96). Major research on WWOX in breast cancer are listed in Table 1. Figure 1 depicts crucial nodes of differential WWOX molecular functions and its clinical implications.

**Table 1:** Historical and contextual advances in breast cancer *WWOX* research.

**WWOX role or association**		**References**
DNA damage response and genome instability	“Fhit and Wwox loss-associated genome instability: A genome caretaker one-two punch”	Schrock et al. (99)
	“Wwox-Brca1 interaction: role in DNA repair pathway choice”	Schrock et al. (64)
	WWOX modulates the ATR-mediated DNA damage checkpoint response	Abu-Odeh et al. (63)
	“WWOX, the common fragile site FRA16D gene product, regulates ATM activation and the DNA damage response”	Abu-Odeh et al. (61)
	“Aberrant expression of DNA damage response proteins is associated with breast cancer subtype and clinical features”	Guler et al. (60)
Bone metastasis from breast carcinoma	“Epigenetic regulation of HGF/Met receptor axis is critical for the outgrowth of bone metastasis from breast carcinoma”	Bendinelli et al. (100)
	“Functions and epigenetic regulation of Wwox in bone metastasis from breast Carcinoma: Comparison with primary tumors”	Maroni et al. (95)
	“HGF and TGFβ1 differently influenced Wwox regulatory function on Twist program for mesenchymal-epithelial transition in bone metastatic vs. parental breast carcinoma cells”	Bendinelli et al. (94)
	“Hypoxia induced E-cadherin involving regulators of Hippo pathway due to HIF-1α stabilization/nuclear translocation in bone metastasis from breast carcinoma”	Maroni et al. (93)
	“Hypoxia inducible factor-1 is activated by transcriptional co-activator with PDZ-binding motif (TAZ) vs. WWdomain-containing oxidoreductase (WWOX) in hypoxic microenvironment of bone metastasis from breast cancer”	Bendinelli et al. (101)
	“Bone metastatic process of breast cancer involves methylation state affecting E-cadherin expression through TAZ and WWOX nuclear effectors”	Matteucci et al. (92)
Glucose metabolism	“Tumor suppressor WWOX regulates glucose metabolism via HIF1α modulation”	Abu-Remaileh et al. (96)
	“WWOX loss activates aerobic glycolysis”	Abu-Remaileh et al. (102)
Mammary branching morphogenesis	“Characterization of WWOX inactivation in murine mammary gland development”	Abdeen et al. (103)
	“Conditional Wwox deletion in mouse mammary gland by means of two Cre recombinase approaches”	Ferguson et al.(72)
	“WWOX, the tumor suppressor gene affected in multiple cancers”	Lewandowska et al. (68)
WNT signaling pathways	“Conditional Wwox deletion in mouse mammary gland by means of two Cre recombinase approaches”	Ferguson et al. (72)
	“Inhibition of the Wnt/beta-catenin pathway by the WWOX tumor suppressor protein”	Bouteille et al. (42)
Transcription regulation	“The cancer gene WWOX behaves as an inhibitor of SMAD3 transcriptional activity via direct binding”	Ferguson et al. (44)
	“WW domain-containing proteins, WWOX and YAP, compete for interaction with ErbB4 and modulate its transcriptional function”	Aqeilan et al. (46)
	“Physical and functional interactions between the Wwox tumor suppressor protein and the AP-2gamma transcription factor”	Aqeilan et al. (40)
Tamoxifen resistance in BC patients	“Wwox expression may predict benefit from adjuvant tamoxifen in randomized breast cancer patients”	Göthlin et al. (91)
	“Wwox inactivation enhances mammary tumorigenesis”	Abdeen et al. (71)
	“Wwox and Ap2γ expression levels predict tamoxifen response”	Guler et al. (90)
Lymph node metastasis in BC patients	“Aberrant expression of DNA damage response proteins is associated with breast cancer subtype and clinical features”	Guler et al. (60)
Prognostic marker in BC patients	“The prognostic significance of WWOX expression in patients with breast cancer and its association with the basal-like phenotype”	Wang et al. (104)
	“Breast cancer relapse prediction based on multi-gene RT-PCR algorithm”	Pluciennik et al. (87)
	“Association of Wwox with ErbB4 in breast cancer”	Aqeilan et al. (105)
	“WWOX–the FRA16D cancer gene: expression correlation with breast cancer progression and prognosis”	Pluciennik et al. (88)
ER correlation in breast carcinomas	“Fragile histidine triad protein, WW domain-containing oxidoreductase protein Wwox, and activator protein2γ expression levels correlate with basal phenotype in breast cancer”	Guler et al. (89)
	“Frequent loss of WWOX expression in breast cancer: correlation with estrogen receptor status”	Nunez et al. (85)
	“The fragile genes FHIT and WWOX are inactivated coordinately in invasive breast carcinoma”	Guler et al. (84)
Hypermethylation	“Association between CpG island methylation of the WWOX gene and its expression in breast cancers”	Wang et al. (75)
	“Inhibition of breast cancer cell growth *in vitro* and *in vivo*: effect of restoration of Wwox expression”	Iliopoulos et al. (69)
	“Fragile genes as biomarkers: epigenetic control of WWOX and FHIT in lung, breast and bladder cancer”	Iliopoulos et al. (74)
Loss of expression	“Inhibition of breast cancer cell growth *in vitro* and *in vivo*: effect of restoration of Wwox expression”	Iliopoulos et al. (69)
	“The fragile genes FHIT and WWOX are inactivated coordinately in invasive breast carcinoma”	Guler et al. (84)
	“WWOX, the FRA16D gene, behaves as a suppressor of tumor growth”	Bednarek et al. (31)
	“Deletion map of chromosome 16q in ductal carcinoma *in situ* of the breast: refining a putative tumor suppressor gene region”	Chen et al. (9)
LOH	“WWOX: a candidate tumor suppressor gene involved in multiple tumor types”	Paige et al. (34)
	“WWOX, the FRA16D gene, behaves as a suppressor of tumor growth”	Bednarek et al. (31)
	“WWOX, a novel WW domain-containing protein mapping to human chromosome 16q23.3–24.1, a region frequently affected in breast cancer”	Bednarek et al. (1)
	“Construction of a high-resolution physical and transcription map of chromosome 16q24.3: a region of frequent loss of heterozygosity in sporadic breast cancer”	Whitmore et al. (10)
	“Deletion map of chromosome 16q in ductal carcinoma *in situ* of the breast: refining a putative tumor suppressor gene region”	Chen et al. (9)

## Summary and future perspectives

Initially, when the *WWOX* gene was discovered, it seemed almost certain that it was related to the classical tumor suppressor gene. Downregulation of WWOX expression was found to be associated with more advanced tumors, higher aggressiveness, and poorer disease-free and overall patient survival, not only in breast cancer but in the majority of investigated neoplasms. However, despite evidence from clinical outcomes and *in vivo* and *in vitro* models supporting its tumor suppressor function, it was found that WWOX does not act as a standard tumor suppressor gene. In various cancer types, it is more common to observe low levels of Wwox than none at all, which does not fit Knudson's two-hit hypothesis. In fact its haploinsufficiency is prevalent for WWOX inactivation, and inactivation of both alleles or missense mutations are very rare.

WWOX possessing a classical SDR domain turned out to take part in steroid and bone metabolism. The *WWOX* gene is in fact highly expressed in hormone-dependent tissues, where it might be involved in the regulation of metabolic steroids. Reduced or lost expression of WWOX resulted in the development of metabolic diseases, and Wwox KO mice were found to die early prematurely from multiple physiological defects before any tumors developed.

Two WW domains of Wwox are responsible for its interactions. Although several transcription factors have been identified amongst WWOX partners, various Wwox interactors have been found to play roles in aerobic metabolism. Indeed, the contribution of Wwox to pathways involving aerobic metabolism and oxidative stress are well-documented, which provides further evidence for the non-classical tumor suppressor characteristics of WWOX. Its ability to facilitate the circumvention of mitochondrial damage-induced glycolysis (Warburg effect) was proposed as a possible mechanism for its tumor suppressor activity.

Loss or dysregulation of WWOX expression leads not only to tumorigenesis and cancer progression, but also genomic instability and resistance to therapy. This way, WWOX can be used as a potent marker of prognostic and clinical outcome. Wwox expression might also serve as a significant predictor of response to radiotherapy and chemotherapy.

WWOX is involved in many signaling pathways for regulating cell apoptosis, autophagy, differentiation and metabolism. Mutations and deletions completely silencing *WWOX* expression are very rare and most often we observe abrogation of its function through lowered WWOX protein synthesis and haploinsufficiency. Therefore, due to multifunctional nature of WWOX there is very difficult to justify prevalent dysfunction in a specific tumor type. Final result of *WWOX* gene silencing depends also on abrogation of function of its partner proteins and appropriate protein network. That suggests that while in breast cancer we observe around 70% loss of heterozygosity of WWOX gene and significant expression reduction final effect of WWOX abrogation depends on topology of multiprotein network associated with WWOX protein-protein interaction that may be differentiated in tumor subtypes.

## Author contributions

KP, EP, and AB contributed to the design and to the writing of the manuscript.

### Conflict of interest statement

The authors declare that the research was conducted in the absence of any commercial or financial relationships that could be construed as a potential conflict of interest.

## References

[1] Bednarek AK Laflin KJ Daniel RL Liao Q Hawkins KA Aldaz CM. WWOX, a novel WW domain-containing protein mapping to human chromosome 16q23.3-24.1, a region frequently affected in breast cancer. Cancer Res. (2000) 60:2140–5. 10786676

[2] Carter BS Ewing CM Ward WS Treiger BF Aalders TW Schalken JA. Allelic loss of chromosomes 16q and 10q in human prostate cancer. Proc Natl Acad Sci USA. (1990) 87:8751–5. 10.1073/pnas.87.22.8751 1978938 PMC55037

[3] Cheng I Levin AM Tai YC Plummer S Chen GK Neslund-Dudas C. Copy number alterations in prostate tumors and disease aggressiveness. Genes Chromosomes Cancer (2012) 51:66–76. 10.1002/gcc.20932 21965145 PMC3209417

[4] Nishida N Fukuda Y Kokuryu H Sadamoto T Isowa G Honda K. Accumulation of allelic loss on arms of chromosomes 13q, 16q and 17p in the advanced stages of human hepatocellular carcinoma. Int J Cancer (1992) 51:862–8. 10.1002/ijc.2910510605 1322376

[5] Aderca I Moser CD Veerasamy M Bani-Hani AH Bonilla-Guerrero R Ahmed K. The JNK inhibitor SP60(0129)enhances apoptosis of HCC cells induced by the tumor suppressor WWOX. J Hepatol. (2008) 49:373–83. 10.1016/j.jhep.2008.05.015 18620777 PMC2574998

[6] Aqeilan RI Kuroki T Pekarsky Y Albagha O Trapasso F Baffa R. Loss of WWOX expression in gastric carcinoma. Clin Cancer Res. (2004) 10:3053–8. 10.1158/1078-0432.CCR-03-0594 15131042

[7] Yakicier MC Legoix P Vaury C Gressin L Tubacher E Capron F. Identification of homozygous deletions at chromosome 16q23 in aflatoxin B1 exposed hepatocellular carcinoma. Oncogene (2001) 20:5232–8. 10.1038/sj.onc.1204674 11526514

[8] Iwabuchi H Sakamoto M Sakunaga H Ma YY Carcangiu ML Pinkel D. Genetic analysis of benign, low-grade, and high-grade ovarian tumors. Cancer Res. (1995) 55:6172–80. 8521410

[9] Chen T Sahin A Aldaz CM. Deletion map of chromosome 16q in ductal carcinoma *in situ* of the breast: refining a putative tumor suppressor gene region. Cancer Res. (1996) 56:5605–9. 8971163

[10] Whitmore SA Crawford J Apostolou S Eyre H Baker E Lower KM. Construction of a high-resolution physical and transcription map of chromosome 16q24.3: a region of frequent loss of heterozygosity in sporadic breast cancer. Genomics (1998) 50:1–8. 10.1006/geno.1998.5316 9628816

[11] Wang M Gu J Wang Y Gong B. Loss of WWOX expression in human extrahepatic cholangiocarcinoma. J Cancer Res Clin Oncol. (2009) 135:39–44. 10.1007/s00432-008-0449-4 18629536 PMC12160241

[12] Kuroki T Trapasso F Shiraishi T Alder H Mimori K Mori M. Genetic alterations of the tumor suppressor gene WWOX in esophageal squamous cell carcinoma. Cancer Res. (2002) 62:2258–60. 11956080

[13] Nancarrow DJ Handoko HY Smithers BM Gotley DC Drew PA Watson DI. Genome-wide copy number analysis in esophageal adenocarcinoma using high-density single-nucleotide polymorphism arrays. Cancer Res. (2008) 68:4163–72. 10.1158/0008-5472.CAN-07-6710 18519675

[14] Yendamuri S Kuroki T Trapasso F Henry AC Dumon KR Huebner K. WW domain containing oxidoreductase gene expression is altered in non-small cell lung cancer. Cancer Res. (2003) 63:878–81. 12591741

[15] Nakayama S Semba S Maeda N Aqeilan RI Huebner K Yokozaki H. Role of the WWOX gene, encompassing fragile region FRA16D, in suppression of pancreatic carcinoma cells. Cancer Sci. (2008) 99:1370–6. 10.1111/j.1349-7006.2008.00841.x 18460020 PMC11159152

[16] Kuroki T Yendamuri S Trapasso F Matsuyama A Aqeilan RI Alder H. The tumor suppressor gene WWOX at FRA16D is involved in pancreatic carcinogenesis. Clin Cancer Res. (2004) 10:2459–65. 10.1158/1078-0432.CCR-03-0096 15073125

[17] Jenner MW Leone PE Walker BA Ross FM Johnson DC Gonzalez D. Gene mapping and expression analysis of 16q loss of heterozygosity identifies WWOX and CYLD as being important in determining clinical outcome in multiple myeloma. Blood (2007) 110:3291–300. 10.1182/blood-2007-02-075069 17609426

[18] Sbrana I Veroni F Nieri M Puliti A Barale R. Chromosomal fragile sites FRA3B and FRA16D show correlated expression and association with failure of apoptosis in lymphocytes from patients with thyroid cancer. Genes Chromosomes Cancer (2006) 45:429–36. 10.1002/gcc.20305 16419058

[19] Kosla K Pluciennik E Kurzyk A Jesionek-Kupnicka D Kordek R Potemski P. Molecular analysis of WWOX expression correlation with proliferation and apoptosis in glioblastoma multiforme. J Neurooncol. (2011) 101:207–13. 10.1007/s11060-010-0254-1 20535528 PMC2996532

[20] Skotnicka-Klonowicz G Rieske P Bartkowiak J Szymik-Kantorowicz S Daszkiewicz P Debiec-Rychter M. 16q heterozygosity loss in Wilms' tumour in children and its clinical importance. Eur J Surg Oncol. (2000) 26:61–6. 10.1053/ejso.1999.0742 10718182

[21] Donati V Fontanini G Dell'Omodarme M Prati MC Nuti S Lucchi M. WWOX expression in different histologic types and subtypes of non-small cell lung cancer. Clin Cancer Res Off J Am Assoc Cancer Res. (2007) 13:884–91. 10.1158/1078-0432.CCR-06-2016 17289881

[22] Bloomston M Kneile J Butterfield M Dillhoff M Muscarella P Ellison EC. Coordinate loss of fragile gene expression in pancreatobiliary cancers: correlations among markers and clinical features. Ann Surg Oncol. (2009) 16:2331–8. 10.1245/s10434-009-0507-4 19434452 PMC2719793

[23] Watson JEV Doggett NA Albertson DG Andaya A Chinnaiyan A van Dekken H. Integration of high-resolution array comparative genomic hybridization analysis of chromosome 16q with expression array data refines common regions of loss at 16q23-qter and identifies underlying candidate tumor suppressor genes in prostate cancer. Oncogene (2004) 23:3487–94. 10.1038/sj.onc.1207474 15007382

[24] Nunez MI Rosen DG Ludes-Meyers JH Abba MC Kil H Page R. WWOX protein expression varies among ovarian carcinoma histotypes and correlates with less favorable outcome. BMC Cancer (2005) 5:64. 10.1186/1471-2407-5-64 15982416 PMC1173095

[25] Dias EP Pimenta FJ Sarquis MS Dias Filho MA Aldaz CM Fujii JB. Association between decreased WWOX protein expression and thyroid cancer development. Thyroid Off J Am Thyroid Assoc. (2007) 17:1055–9. 10.1089/thy.2007.0232 18047428 PMC4150466

[26] Ramos D Abba M López-Guerrero JA Rubio J Solsona E Almenar S. Low levels of WWOX protein immunoexpression correlate with tumour grade and a less favourable outcome in patients with urinary bladder tumours. Histopathology (2008) 52:831–9. 10.1111/j.1365-2559.2008.03033.x 18452537 PMC4151645

[27] Nunez MI Ludes-Meyers J Aldaz CM. WWOX protein expression in normal human tissues. J Mol Histol. (2006) 37:115–25. 10.1007/s10735-006-9046-5 16941225 PMC4144810

[28] Asmann YW Necela BM Kalari KR Hossain A Baker TR Carr JM. Detection of redundant fusion transcripts as biomarkers or disease-specific therapeutic targets in breast cancer. Cancer Res. (2012) 72:1921–8. 10.1158/0008-5472.CAN-11-3142 22496456

[29] Duff MO Olson S Wei X Garrett SC Osman A Bolisetty M. Genome-wide identification of zero nucleotide recursive splicing in Drosophila. Nature (2015) 521:376–9. 10.1038/nature14475 25970244 PMC4529404

[30] Fagerberg L Hallström BM Oksvold P Kampf C Djureinovic D Odeberg J. Analysis of the human tissue-specific expression by genome-wide integration of transcriptomics and antibody-based proteomics. Mol Cell Proteomics (2014) 13:397–406. 10.1074/mcp.M113.035600 24309898 PMC3916642

[31] Bednarek AK Keck-Waggoner CL Daniel RL Laflin KJ Bergsagel PL Kiguchi K. WWOX, the FRA16D gene, behaves as a suppressor of tumor growth. Cancer Res. (2001) 61:8068–73. 11719429

[32] Driouch K Prydz H Monese R Johansen H Lidereau R Frengen E. Alternative transcripts of the candidate tumor suppressor gene, WWOX, are expressed at high levels in human breast tumors. Oncogene (2002) 21:1832–40. 10.1038/sj.onc.1205273 11896615

[33] Watanabe A Hippo Y Taniguchi H Iwanari H Yashiro M Hirakawa K. An opposing view on WWOX protein function as a tumor suppressor. Cancer Res. (2003) 63:8629–33. 14695174

[34] Paige AJ Taylor KJ Taylor C Hillier SG Farrington S Scott D. WWOX: a candidate tumor suppressor gene involved in multiple tumor types. Proc Natl Acad Sci USA (2001) 98:11417–22. 10.1073/pnas.191175898 11572989 PMC58744

[35] McDonald CB Buffa L Bar-Mag T Salah Z Bhat V Mikles DC. Biophysical basis of the binding of WWOX tumor suppressor to WBP1 and WBP2 adaptors. J Mol Biol. (2012) 422:58–74. 10.1016/j.jmb.2012.05.015 22634283 PMC3412936

[36] Schuchardt BJ Mikles DC Bhat V McDonald CB Sudol M Farooq A. Allostery mediates ligand binding to WWOX tumor suppressor via a conformational switch. J Mol Recognit. (2015) 28:220–31. 10.1002/jmr.2419 25703206 PMC4376589

[37] Abu-Odeh M Bar-Mag T Huang H Kim T Salah Z Abdeen SK. Characterizing WW domain interactions of tumor suppressor WWOX reveals its association with multiprotein networks. J Biol Chem. (2014) 289:8865–80. 10.1074/jbc.M113.506790 24550385 PMC3979411

[38] Schuchardt BJ Bhat V Mikles DC McDonald CB Sudol M Farooq A. Molecular origin of the binding of WWOX tumor suppressor to ErbB4 receptor tyrosine kinase. Biochemistry (2013) 52:9223–36. 10.1021/bi400987k 24308844 PMC3906126

[39] Salah Z Aqeilan R Huebner K. WWOX gene and gene product: tumor suppression through specific protein interactions. Future Oncol Lond Engl. (2010) 6:249–59. 10.2217/fon.09.152 20146584 PMC2832309

[40] Aqeilan RI Palamarchuk A Weigel RJ Herrero JJ Pekarsky Y Croce CM. Physical and functional interactions between the Wwox tumor suppressor protein and the AP-2gamma transcription factor. Cancer Res. (2004) 64:8256–61. 10.1158/0008-5472.CAN-04-2055 15548692

[41] Gaudio E Palamarchuk A Palumbo T Trapasso F Pekarsky Y Croce CM. Physical association with WWOX suppresses c-Jun transcriptional activity. Cancer Res. (2006) 66:11585–9. 10.1158/0008-5472.CAN-06-3376 17178850

[42] Bouteille N Driouch K Hage PE Sin S Formstecher E Camonis J. Inhibition of the Wnt/beta-catenin pathway by the WWOX tumor suppressor protein. Oncogene (2009) 28:2569–80. 10.1038/onc.2009.120 19465938

[43] Aqeilan RI Hassan MQ de Bruin A Hagan JP Volinia S Palumbo T. The WWOX tumor suppressor is essential for postnatal survival and normal bone metabolism. J Biol Chem. (2008) 283:21629–39. 10.1074/jbc.M800855200 18487609 PMC2490770

[44] Ferguson BW Gao X Zelazowski MJ Lee J Jeter CR Abba MC. The cancer gene WWOX behaves as an inhibitor of SMAD3 transcriptional activity via direct binding. BMC Cancer (2013) 13:593. 10.1186/1471-2407-13-593 24330518 PMC3871008

[45] Xiong A Wei L Ying M Wu H Hua J Wang Y. Wwox suppresses breast cancer cell growth through modulation of the hedgehog-GLI1 signaling pathway. Biochem Biophys Res Commun. (2014) 443:1200–5. 10.1016/j.bbrc.2013.12.133 24393846

[46] Aqeilan RI Donati V Palamarchuk A Trapasso F Kaou M Pekarsky Y. WW domain-containing proteins, WWOX and YAP, compete for interaction with ErbB-4 and modulate its transcriptional function. Cancer Res. (2005) 65:6764–72. 10.1158/0008-5472.CAN-05-1150 16061658

[47] Jin C Ge L Ding X Chen Y Zhu H Ward T. PKA-mediated protein phosphorylation regulates ezrin-WWOX interaction. Biochem Biophys Res Commun. (2006) 341:784–91. 10.1016/j.bbrc.2006.01.023 16438931

[48] Bruce B Khanna G Ren L Landberg G Jirström K Powell C. Expression of the cytoskeleton linker protein ezrin in human cancers. Clin Exp Metastasis (2007) 24:69–78. 10.1007/s10585-006-9050-x 17370041

[49] Chang N-S Doherty J Ensign A Schultz L Hsu LJ Hong Q. WOX1 is essential for tumor necrosis factor-, UV light-, staurosporine-, and p53-mediated cell death, and its tyrosine 33-phosphorylated form binds and stabilizes serine 46-phosphorylated p53. J Biol Chem. (2005) 280:43100–8. 10.1074/jbc.M505590200 16219768

[50] Jörnvall H Persson B Krook M Atrian S Gonzàlez-Duarte R Jeffery J. Short-chain dehydrogenases/reductases (SDR). Biochemistry (1995) 34:6003–13. 10.1021/bi00018a001 7742302

[51] Kallberg Y Oppermann U Jörnvall H Persson B. Short-chain dehydrogenases/reductases (SDRs). Eur J Biochem. (2002) 269:4409–17. 10.1046/j.1432-1033.2002.03130.x 12230552

[52] Duax WL Ghosh D. Structure and function of steroid dehydrogenases involved in hypertension, fertility, and cancer. Steroids (1997) 62:95–100. 10.1016/S0039-128X(96)00166-3 9029722

[53] Kallberg Y Oppermann U Jörnvall H Persson B. Short-chain dehydrogenase/reductase (SDR) relationships: a large family with eight clusters common to human, animal, and plant genomes. Protein Sci Publ Protein Soc. (2002) 11:636–41. 10.1110/ps.26902 11847285 PMC2373483

[54] Marijanovic Z Laubner D Moller G Gege C Husen B Adamski J. Closing the gap: identification of human 3-ketosteroid reductase, the last unknown enzyme of mammalian cholesterol biosynthesis. Mol Endocrinol Baltim Md. (2003) 17:1715–25. 10.1210/me.2002-0436 12829805

[55] Sałuda-Gorgul A Seta K Nowakowska M Bednarek AK. WWOX oxidoreductase–substrate and enzymatic characterization. Z Naturforschung C (2011) 66:73–82. 10.1515/znc-2011-1-210 21476439

[56] Chang N-S Schultz L Hsu L-J Lewis J Su M Sze C-I. 17beta-Estradiol upregulates and activates WOX1/WWOXv1 and WOX2/WWOXv2 *in vitro*: potential role in cancerous progression of breast and prostate to a premetastatic state *in vivo*. Oncogene (2005) 24:714–23. 10.1038/sj.onc.1208124 15580310

[57] Aqeilan RI Hagan JP de Bruin A Rawahneh M Salah Z Gaudio E. Targeted ablation of the WW domain-containing oxidoreductase tumor suppressor leads to impaired steroidogenesis. Endocrinology (2009) 150:1530–5. 10.1210/en.2008-1087 18974271 PMC2654736

[58] Lee JC Weissglas-Volkov D Kyttälä M Dastani Z Cantor RM Sobel EM. WW-domain-containing oxidoreductase is associated with low plasma HDL-C levels. Am J Hum Genet. (2008) 83:180–92. 10.1016/j.ajhg.2008.07.002 18674750 PMC2495060

[59] Iatan I Choi HY Ruel I Reddy MVPL Kil H Lee J. The WWOX gene modulates high-density lipoprotein and lipid metabolism. Circ Cardiovasc Genet. (2014) 7:491–504. 10.1161/CIRCGENETICS.113.000248 24871327 PMC4315188

[60] Guler G Himmetoglu C Jimenez RE Geyer SM Wang WP Costinean S. Aberrant expression of DNA damage response proteins is associated with breast cancer subtype and clinical features. Breast Cancer Res Treat. (2011) 129:421–32. 10.1007/s10549-010-1248-6 21069451 PMC3677189

[61] Abu-Odeh M Salah Z Herbel C Hofmann TG Aqeilan RI. WWOX, the common fragile site FRA16D gene product, regulates ATM activation and the DNA damage response. Proc Natl Acad Sci USA. (2014) 111:E4716–25. 10.1073/pnas.1409252111 25331887 PMC4226089

[62] Aqeilan RI Abu-Remaileh M Abu-Odeh M. The common fragile site FRA16D gene product WWOX: roles in tumor suppression and genomic stability. Cell Mol Life Sci. (2014) 71:4589–99. 10.1007/s00018-014-1724-y 25245215 PMC11113097

[63] Abu-Odeh M Hereema NA Aqeilan RI. WWOX modulates the ATR-mediated DNA damage checkpoint response. Oncotarget (2016) 7:4344–55. 10.18632/oncotarget.6571 26675548 PMC4826209

[64] Schrock MS Batar B Lee J Druck T Ferguson B Cho JH. Wwox-Brca1 interaction: role in DNA repair pathway choice. Oncogene (2017) 36:2215–27. 10.1038/onc.2016.389 27869163 PMC5398941

[65] Fabbri M Iliopoulos D Trapasso F Aqeilan RI Cimmino A Zanesi N. WWOX gene restoration prevents lung cancer growth *in vitro* and *in vivo*. Proc Natl Acad Sci USA. (2005) 102:15611–6. 10.1073/pnas.0505485102 16223882 PMC1266103

[66] Qin HR Iliopoulos D Semba S Fabbri M Druck T Volinia S. A role for the WWOX gene in prostate cancer. Cancer Res. (2006) 66:6477–81. 10.1158/0008-5472.CAN-06-0956 16818616

[67] Gourley C Paige AJW Taylor KJ Ward C Kuske B Zhang J. WWOX gene expression abolishes ovarian cancer tumorigenicity *in vivo* and decreases attachment to fibronectin via integrin alpha3. Cancer Res. (2009) 69:4835–42. 10.1158/0008-5472.CAN-08-2974 19458077

[68] Lewandowska U Zelazowski M Seta K Byczewska M Pluciennik E Bednarek AK. WWOX, the tumour suppressor gene affected in multiple cancers. J Physiol Pharmacol. c. (2009) 60 (Suppl. 1):47–56. 19609013

[69] Iliopoulos D Fabbri M Druck T Qin HR Han SY Huebner K. Inhibition of breast cancer cell growth *in vitro* and *in vivo*: effect of restoration of Wwox expression. Clin Cancer Res. (2007) 13:268–74. 10.1158/1078-0432.CCR-06-2038 17200365

[70] Xiong Z Hu S Wang Z. Cloning of WWOX gene and its growth-inhibiting effects on ovarian cancer cells. J Huazhong Univ Sci Technol Med Sci. (2010) 30:365–9. 10.1007/s11596-010-0358-z 20556583

[71] Abdeen SK Salah Z Maly B Smith Y Tufail R Abu-Odeh M. Wwox inactivation enhances mammary tumorigenesis. Oncogene (2011) 30:3900–6. 10.1038/onc.2011.115 21499303

[72] Ferguson BW Gao X Kil H Lee J Benavides F Abba MC. Conditional Wwox deletion in mouse mammary gland by means of two Cre recombinase approaches. PLoS ONE (2012) 7:e36618. 10.1371/journal.pone.0036618 22574198 PMC3344920

[73] Aldaz CM Ferguson BW Abba MC. WWOX at the crossroads of cancer, metabolic syndrome related traits and CNS pathologies. Biochim Biophys Acta (2014) 1846:188–200. 10.1016/j.bbcan.2014.06.001 24932569 PMC4151823

[74] Iliopoulos D Guler G Han S-Y Johnston D Druck T McCorkell KA. Fragile genes as biomarkers: epigenetic control of WWOX and FHIT in lung, breast and bladder cancer. Oncogene (2005) 24:1625–33. 10.1038/sj.onc.1208398 15674328

[75] Wang X Chao L Jin G Ma G Zang Y Sun J. Association between CpG island methylation of the WWOX gene and its expression in breast cancers. Tumour Biol J Int Soc Oncodev Biol Med. (2009) 30:8–14. 10.1159/000197911 19188760

[76] Ekizoglu S Muslumanoglu M Dalay N Buyru N. Genetic alterations of the WWOX gene in breast cancer. Med Oncol Northwood Lond Engl. (2012) 29:1529–35. 10.1007/s12032-011-0080-0 21983861

[77] Finnis M Dayan S Hobson L Chenevix-Trench G Friend K Ried K. Common chromosomal fragile site FRA16D mutation in cancer cells. Hum Mol Genet. (2005) 14:1341–9. 10.1093/hmg/ddi144 15814586

[78] Mahajan NP Whang YE Mohler JL Earp HS. Activated tyrosine kinase Ack1 promotes prostate tumorigenesis: role of Ack1 in polyubiquitination of tumor suppressor Wwox. Cancer Res. (2005) 65:10514–23. 10.1158/0008-5472.CAN-05-1127 16288044

[79] Yan J Zhang M Zhang J Chen X Zhang X. Helicobacter pylori infection promotes methylation of WWOX gene in human gastric cancer. Biochem Biophys Res Commun. (2011) 408:99–102. 10.1016/j.bbrc.2011.03.127 21466786

[80] Nakayama S Semba S Maeda N Matsushita M Kuroda Y Yokozaki H. Hypermethylation-mediated reduction of WWOX expression in intraductal papillary mucinous neoplasms of the pancreas. Br J Cancer (2009) 100:1438–43. 10.1038/sj.bjc.6604986 19352382 PMC2694421

[81] Baykara O Demirkaya A Kaynak K Tanju S Toker A Buyru N. WWOX gene may contribute to progression of non-small-cell lung cancer (NSCLC). Tumour Biol J Int Soc Oncodev Biol Med. (2010) 31:315–20. 10.1007/s13277-010-0039-3 20480411

[82] Fabbri M Garzon R Cimmino A Liu Z Zanesi N Callegari E. MicroRNA-29 family reverts aberrant methylation in lung cancer by targeting DNA methyltransferases 3A and 3B. Proc Natl Acad Sci USA. (2007) 104:15805–10. 10.1073/pnas.0707628104 17890317 PMC2000384

[83] Ludes-Meyers JH Bednarek AK Popescu NC Bedford M Aldaz CM. WWOX, the common chromosomal fragile site, FRA16D, cancer gene. Cytogenet Genome Res. (2003) 100:101–10. 10.1159/000072844 14526170 PMC4150470

[84] Guler G Uner A Guler N Han S-Y Iliopoulos D Hauck WW. The fragile genes FHIT and WWOX are inactivated coordinately in invasive breast carcinoma. Cancer (2004) 100:1605–14. 10.1002/cncr.20137 15073846

[85] Nunez MI Ludes-Meyers J Abba MC Kil H Abbey NW Page RE. Frequent loss of WWOX expression in breast cancer: correlation with estrogen receptor status. Breast Cancer Res Treat. (2005) 89:99–105. 10.1007/s10549-004-1474-x 15692750 PMC4145848

[86] Guler G Uner A Guler N Han SY Iliopoulos D McCue P. Concordant loss of fragile gene expression early in breast cancer development. Pathol Int. (2005) 55:471–8. 10.1111/j.1440-1827.2005.01855.x 15998374

[87] Pluciennik E Krol M Nowakowska M Kusinska R Potemski P Kordek R. Breast cancer relapse prediction based on multi-gene RT-PCR algorithm. Med Sci Monit Int Med J Exp Clin Res. (2010) 16:CR132–136. 20190683

[88] Płuciennik E Kusinska R Potemski P Kubiak R Kordek R Bednarek AK. WWOX–the FRA16D cancer gene: expression correlation with breast cancer progression and prognosis. Eur J Surg Oncol. (2006) 32:153–7. 10.1016/j.ejso.2005.11.002 16360296

[89] Guler G Huebner K Himmetoglu C Jimenez RE Costinean S Volinia S. Fragile histidine triad protein, WW domain-containing oxidoreductase protein Wwox, and activator protein 2gamma expression levels correlate with basal phenotype in breast cancer. Cancer (2009) 115:899–908. 10.1002/cncr.24103 19130459 PMC2640223

[90] Guler G Iliopoulos D Guler N Himmetoglu C Hayran M Huebner K. Wwox and Ap2gamma expression levels predict tamoxifen response. Clin Cancer Res Off J Am Assoc Cancer Res. (2007) 13:6115–21. 10.1158/1078-0432.CCR-07-1282 17947476

[91] Göthlin Eremo A Wegman P Stål O Nordenskjöld B Fornander T Wingren S. Wwox expression may predict benefit from adjuvant tamoxifen in randomized breast cancer patients. Oncol Rep. (2013) 29:1467–74. 10.3892/or.2013.2261 23381945

[92] Matteucci E Maroni P Luzzati A Perrucchini G Bendinelli P Desiderio MA. Bone metastatic process of breast cancer involves methylation state affecting E-cadherin expression through TAZ and WWOX nuclear effectors. Eur J Cancer (2013) 49:231–44. 10.1016/j.ejca.2012.05.006 22717556

[93] Maroni P Matteucci E Drago L Banfi G Bendinelli P Desiderio MA. Hypoxia induced E-cadherin involving regulators of Hippo pathway due to HIF-1α stabilization/nuclear translocation in bone metastasis from breast carcinoma. Exp Cell Res. (2015) 330:287–99. 10.1016/j.yexcr.2014.10.004 25447306

[94] Bendinelli P Maroni P Matteucci E Desiderio MA. HGF and TGFβ1 differently influenced Wwox regulatory function on twist program for mesenchymal-epithelial transition in bone metastatic versus parental breast carcinoma cells. Mol Cancer (2015) 14:112. 10.1186/s12943-015-0389-y 26041563 PMC4453100

[95] Maroni P Matteucci E Bendinelli P Desiderio MA. Functions and epigenetic regulation of wwox in bone metastasis from breast carcinoma: comparison with primary tumors. Int J Mol Sci. (2017) 18:75. 10.3390/ijms18010075 28045433 PMC5297710

[96] Abu-Remaileh M Aqeilan RI. Tumor suppressor WWOX regulates glucose metabolism via HIF1α modulation. Cell Death Differ. (2014) 21:1805–14. 10.1038/cdd.2014.95 25012504 PMC4211377

[97] Hanahan D Weinberg RA. Hallmarks of cancer: the next generation. Cell (2011) 144:646–74. 10.1016/j.cell.2011.02.013 21376230

[98] Vander Heiden MG Cantley LC Thompson CB. Understanding the Warburg effect: the metabolic requirements of cell proliferation. Science (2009) 324:1029–33. 10.1126/science.1160809 19460998 PMC2849637

[99] Schrock MS Karras JR Guggenbiller MJ Druck T Batar B Huebner K. Fhit and Wwox loss-associated genome instability: a genome caretaker one-two punch. Adv Biol Regul. (2017) 63:167–76. 10.1016/j.jbior.2016.09.008 27773744 PMC7213024

[100] Bendinelli P Maroni P Matteucci E Desiderio MA. Epigenetic regulation of HGF/Met receptor axis is critical for the outgrowth of bone metastasis from breast carcinoma. Cell Death Dis. (2017) 8:e2578. 10.1038/cddis.2016.403 28151481 PMC5386451

[101] Bendinelli P Maroni P Matteucci E Luzzati A Perrucchini G Desiderio MA. Hypoxia inducible factor-1 is activated by transcriptional co-activator with PDZ-binding motif (TAZ) versus WWdomain-containing oxidoreductase (WWOX) in hypoxic microenvironment of bone metastasis from breast cancer. Eur J Cancer (2013) 49:2608–18. 10.1016/j.ejca.2013.03.002 23566416

[102] Abu-Remaileh M Seewaldt VL Aqeilan RI. WWOX loss activates aerobic glycolysis. Mol Cell Oncol. (2015) 2:e965640. 10.4161/23723548.2014.965640 27308416 PMC4904998

[103] Abdeen SK Salah Z Khawaled S Aqeilan RI. Characterization of WWOX inactivation in murine mammary gland development. J Cell Physiol. (2013) 228:1391–6. 10.1002/jcp.24310 23254778

[104] Wang X Chao L Ma G Chen L Zang Y Sun J. The prognostic significance of WWOX expression in patients with breast cancer and its association with the basal-like phenotype. J Cancer Res Clin Oncol. (2011) 137:271–8. 10.1007/s00432-010-0880-20401669 PMC11828298

[105] Aqeilan RI Donati V Gaudio E Nicoloso MS Sundvall M Korhonen A. Association of Wwox with ErbB4 in breast cancer. Cancer Res. (2007) 67:9330–6. 10.1158/0008-5472.CAN-07-2147 17909041

